# Application of “parenchyma-first” approach in laparoscopic living donor left lateral sectionectomy

**DOI:** 10.3389/fsurg.2026.1829650

**Published:** 2026-09-03

**Authors:** Hui Tang, Dongwei Wu, Kaining Zeng, Xiao Feng, Binsheng Fu, Qing Yang, Jia Yao, Yang Yang, Shuhong Yi

**Affiliations:** Department of Hepatic Surgery and Liver Transplantation Center, The Third Affiliated Hospital of Sun Yat-Sen University, Guangzhou, Guangdong, China

**Keywords:** hilar vascular protection, laparoscopic left lateral sectionectomy (LLS), liver diseases, living donor liver transplantation (LDLT), parenchyma-first technique, pediatric liver transplantation

## Abstract

**Objective:**

This study aimed to evaluate the safety and feasibility of a “Parenchyma-First” approach for laparoscopic left lateral sectionectomy (LLS) in living donor hepatectomy, with the primary goal of minimizing vascular injury.

**Methods:**

To avoid premature and excessive dissection of the portal vein and hepatic artery at the first hepatic hilum—which increases the risk of vascular injury—and to prevent torsion or occlusion of these mobilized vessels during parenchymal transection, we adopted a technique that prioritizes liver parenchymal transection before dissecting the left hepatic artery and left portal vein branch. A single-center, retrospective, descriptive study of 46 donors and pediatric recipients (April 2023–June 2025). No control group was included.

**Results:**

Mean donor operative time was 289.7 ± 66.7 min, blood loss 71.5 ± 60.3 mL, and warm ischemia time 297.8 ± 98.6 s. No donor vascular or biliary complications occurred; one donor underwent reoperation for omental bleeding. Among recipients, mortality was 4.3% (2/46) due to recipient-related vascular complications. Vascular or biliary interventions were required in 10.9% (5/46) of recipients. Early allograft dysfunction occurred in 17.4% of recipients.

**Conclusion:**

The “Parenchyma-First” approach for left lateral section graft procurement is technically feasible, provides excellent donor safety by protecting hilar vasculature, and yields acceptable recipient outcomes comparable with international series.

## 1 Introduction

In recent years, international consensus has recognized laparoscopic left lateral sectionectomy (LLS) as the standard procedure for living donor liver transplantation (LDLT) (1). This preference stems from its marked technical advantages over open surgery. Critically, multiple studies have confirmed that grafts obtained laparoscopically show no statistically significant difference in functional recovery or adverse impact on recipient outcomes compared with those from open surgery (2, 3).

The “Hilar-First” approach, a conventional technique in laparoscopic hepatectomy, has been widely adopted for laparoscopic living-donor LLS and is currently used by most transplant centers. However, it still carries certain limitations (4).

The concept of transecting liver parenchyma before hilar dissection (similar to anterior approach) has been reported in open and laparoscopic hepatectomy, but its specific application and anatomical advantages in pediatric LDLT-LLS remain to be clarified. This study does not propose a new surgical philosophy, but presents a tailored technical modification of the “Parenchyma-First” strategy for LDLT-LLS. The core improvement is complete avoidance of early hilar plate lowering and extrahepatic glissonean pedicle skeletonization, which preserves natural vascular support and reduces mechanical trauma to small-caliber hilar structures. This study aimed to summarize the surgical technique and report short-term outcomes of this modified approach.

## 2 Material and methods

### 2.1 Clinical demographics

We retrospectively analyzed clinical data from 46 donors who underwent laparoscopic LDLT-LLS using the “Parenchyma-First” approach at the Liver Surgery and Liver Transplantation Center of the Third Affiliated Hospital of Sun Yat-sen University between April 2023 and June 2025.

### 2.2 Follow ups

Patients were routinely followed up once weekly in the first month after discharge, twice monthly in the second and third months, monthly in the first year, and every 3–4 months thereafter. Median follow-up was 12.6 months for donors and 11.8 months for recipients. Follow-up examinations included blood tests, liver and kidney function, coagulation profiles, and abdominal ultrasound. Chest and abdominal CT scans were performed when clinically indicated. Follow-up continued until recipient death or loss to follow-up, with a cutoff date of July 31, 2025.

### 2.3 Statistical analysis

Statistical analyses were performed using SPSS version 27.0 (IBM, USA). Normality was assessed using the Kolmogorov–Smirnov test. Normally distributed quantitative variables are presented as mean ± standard deviation. Non-normally distributed variables are expressed as median (interquartile range). A two-sided *P*-value < 0.05 was considered statistically significant.

### 2.4 Ethical approval

This study was approved by the Ethics Committee of the Third Affiliated Hospital of Sun Yat-sen University. The requirement for informed consent was waived due to the anonymous and retrospective nature of the study.

## 3 Results

### 3.1 “Parenchyma-first” approach surgical procedure

#### 3.1.1 Patient positioning and trocar placement

The donor was positioned supine in a modified lithotomy position with legs apart and placed in reverse Trendelenburg tilt. A five-port configuration was utilized: a 12-mm camera port was placed infra-umbilically, and the remaining four trocars (two 12-mm and two 5-mm ports) were arranged in a gentle fan-shaped pattern on either side of the midline. The specimen was eventually retrieved through a 6–10 cm transverse suprapubic incision, situated approximately 2 cm above the symphysis pubis.

#### 3.1.2 Intraoperative ultrasound examination

Preoperative CT imaging findings were integrated with intraoperative ultrasonography to reconfirm the anatomy of the portal and hepatic veins and to evaluate donor liver suitability.

#### 3.1.3 Mobilization of perihepatic ligaments

The round ligament, falciform ligament, left coronary ligament, left triangular ligament, and hepatogastric ligament were routinely divided. Particular attention was paid to identifying any accessory left hepatic arteries during division of the hepatogastric ligament. The cranial portion of the ligament of Arantius was transected to fully expose the dorsal aspect of the left hepatic vein root. Complete extrahepatic dissection of the left hepatic vein was not performed, as its root becomes naturally exposed following parenchymal transection.

#### 3.1.4 Indocyanine green (ICG) fluorescence guidance

Indocyanine green (ICG, 0.15 mg/kg) was administered intravenously 8–10 hours before surgery. During the procedure, segment IV was lifted while lowering the hilar plate to clearly visualize the biliary confluence under fluorescence. The transection point on the left hepatic duct was marked with electrocautery approximately 1–2 cm distal to the confluence. The transection line extended along the right side of the falciform ligament (about 1 cm lateral), cranially toward the left hepatic vein root and caudally to the marked bile-duct division point.

#### 3.1.5 Parenchymal transection

Parenchymal transection was carried out with an ultrasonic dissector without prior hilar dissection or inflow control. The transection plane extended cranially to the right side of the left hepatic vein root and caudally to the plane of the left hepatic duct.

#### 3.1.6 Vascular dissection

After parenchymal division, the left (or middle) hepatic artery and the left portal vein branch were carefully dissected from the left border of the hepatoduodenal ligament using blunt technique. Because the left portal vein branch and left hepatic duct remain with the graft, segment IV is deprived of its vascular supply and subsequently undergoes atrophy; therefore, the middle hepatic artery supplying segment IV was carefully preserved with the donor remnant liver, while the left hepatic artery was preserved as long as possible to facilitate recipient anastomosis. Gentle handling was emphasized to avoid intimal injury. The left portal vein was exposed posterior to the artery, and its bifurcation was identified to prevent stenosis. If necessary, the caudate branch was divided to lengthen the portal vein. Excessive dissection toward the right hilum was avoided.

#### 3.1.7 Bile-duct division

After vascular mobilization, the left hepatic duct was re-examined under fluorescence. It was sharply divided (and oversewn on the donor side) or clipped with a Hem-o-lok® before transection, although posterior hilar-plate thickness often limited duct isolation. The graft-side duct was clipped to control bleeding and bile leakage. Preoperative magnetic resonance cholangiopancreatography (MRCP) was used routinely, obviating the need for intraoperative cholangiography. To maximize donor bile-duct safety, transection was frequently biased toward the left side, occasionally resulting in multiple graft ducts; these were managed with meticulous reconstruction in the recipient.

#### 3.1.8 Systemic heparinization

Intravenous heparin (1,000–2,000 IU) was administered before vascular division.

#### 3.1.9 Graft extraction

The suprapubic incision site was prepared by dissecting down to the peritoneum. Vessels were divided in the following sequence:

Artery and portal vein: secured with double Hem-o-lok® ligation.

Left hepatic vein: To prevent anastomotic stenosis in the recipient, a “half-side stapling” technique was employed using a powered endoscopic linear vascular stapler (Ethicon Endo-Surgery, LLC; cartridge PVE35A/VASECR35). The staple cartridge on the caval side was removed before firing, gaining an additional 2–3 mm in length of the left hepatic-vein cuff. On the recipient side, an elliptical or inverted-triangular venoplasty was typically performed for anastomosis. Division was performed under direct vision to ensure complete transection and hemostasis.

The graft was rapidly extracted through the incision for cold perfusion. Pneumoperitoneum was re-established to inspect resection surfaces and vascular stumps. Warm ischemia time—defined as the interval from portal-vein division to cold perfusion—was minimized through swift, coordinated steps.

### 3.2 Donor outcomes

A total of 46 donors were included. Mean age was 31.3 ± 5.46 years, 27 (58.7%) female, mean BMI 21.8 ± 2.8 kg/m^2^. Mean operative time was 289.7 ± 66.7 minutes, mean blood loss 71.5 ± 60.3 mL (maximum 220 mL). No Pringle maneuver was used, and no donor received transfusion.

Mean graft weight was 226.4 ± 38.6 g, LLBWR 0.44 ± 0.08. Reduced-size grafts were used in 25 (54.3%) cases, with mean bench time 11.2 ± 3.6 min. Warm ischemia time was 297.8 ± 98.6 s.

**Figure 1:**
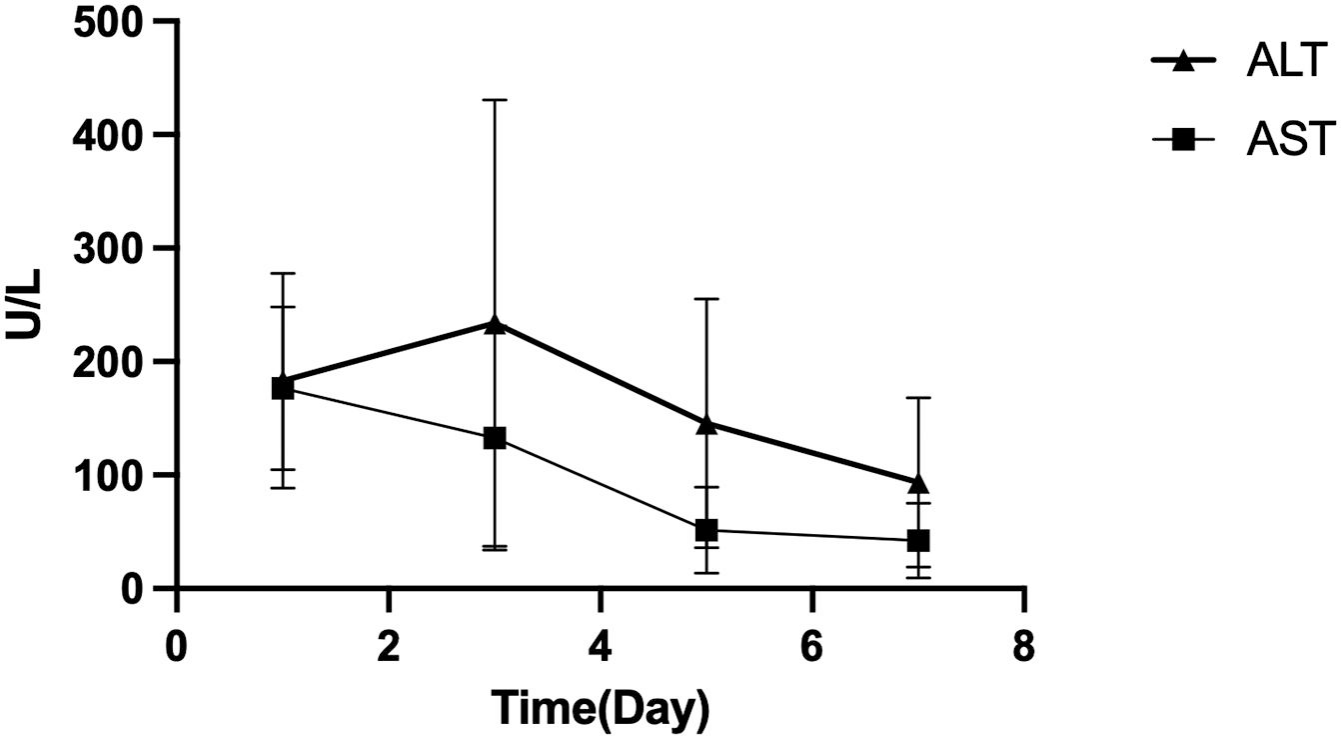
Postoperative liver function profiles in donors by “Parenchyma-First” approach for laparoscopic LLS. ALT, Alanine Aminotransferase; AST, Aspartate Aminotransferase.

Single biliary orifice was present in 24 (52.2%) donors, double in 19 (41.3%), and ≥3 in 3 (6.5%). One donor (2.2%) developed omental hemorrhage and underwent reoperation. No vascular or biliary complications occurred. Median follow-up was 12.6 months. (Tables 1, 2; Figure 1).

**Table 1:** Baseline characteristics of donors.

Donors	*N* = 46
Age (years)	31.3 ± 5.46
Sex (male/female)	19/27
Weight (kg)	59.1 ± 10.7
BMI	21.8 ± 2.8
Preoperative CT left lateral lobe volume (mL)	221.2 ± 80.0
Portal vein branching pattern	
Type I	29 (63.0%)
Type II	15 (32.6%)
Type III	2 (4.3%)
Type IV	0
Type V	0
Hepatic vein drainage pattern classification	
Type I	9 (19.6%)
Type II	37 (80.4%)
Type III	0
Type IV	0

BMI, body mass index; CT, computed tomography.

**Table 2:** Surgical outcomes of donors.

Donors	*N* = 46
Surgical procedure	Left lateral sectionectomy
Operative time (min)	289.7 ± 66.7
Pringle Maneuver	Not performed
Intraoperative blood loss (mL)	71.5 ± 60.3
Graft weight (g)	226.4 ± 38.6
Reduced-size graft	25 (54.3%)
Warm ischemia time (s)	297.8 ± 98.6
Number of hepatic arteries procured	
branch	28 (60.8%)
2 branches	17 (37%)
3 branches	1 (2.2%)
Number of biliary orifices procured	
1 branch	24 (52.2%)
2 branches	19 (41.3%)
3 or 4 branches	3 (6.5%)
Complications	1 (2.2%, omental hemorrhage)
Hospital stays (days)	9.6 ± 2.5

### 3.3 Recipient outcomes

46 pediatric recipients were included. Mean age 13.3 ± 11.6 months, mean weight 7.7 ± 2.5 kg, GRWR 3.0 ± 0.8%. The main etiology was biliary atresia (34, 73.9%). Mean PELD score was 16.1 ± 13.8.

**Figure 2:**
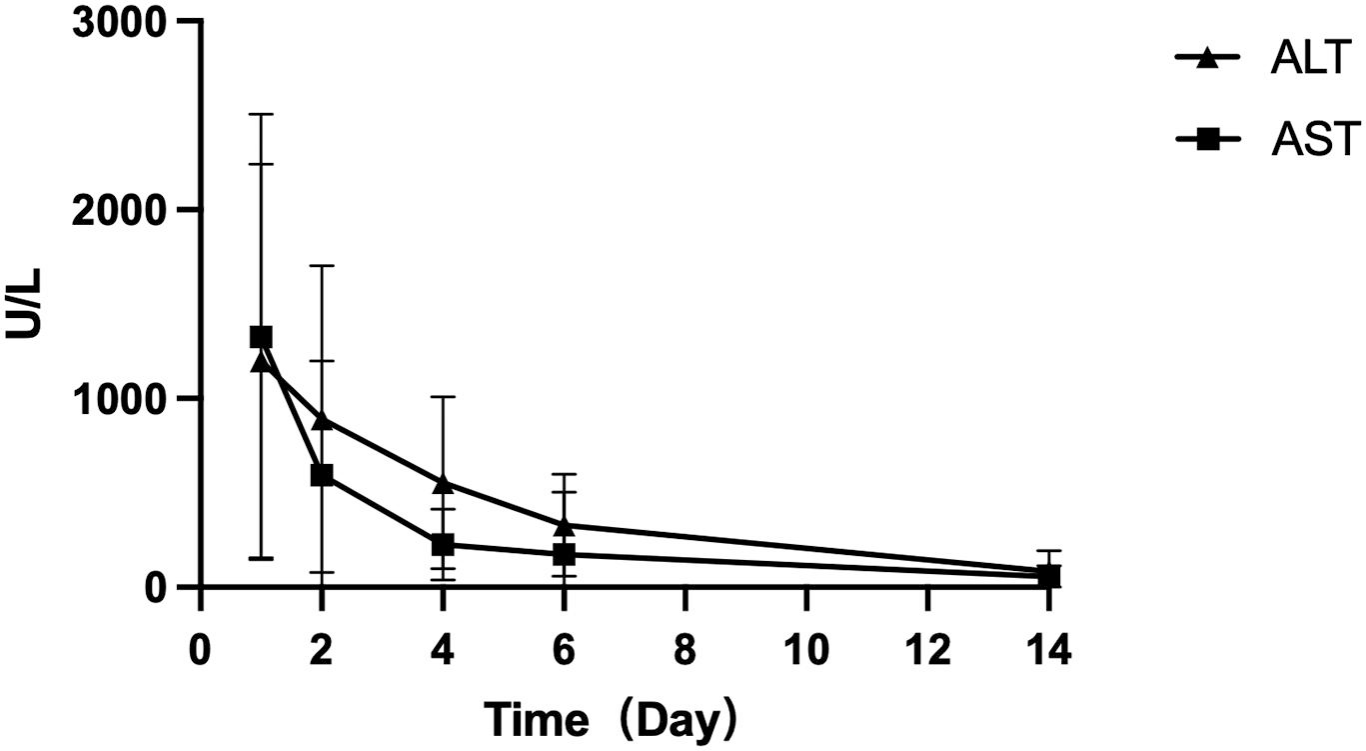
Postoperative liver function profiles in recipients by “Parenchyma-First” approach for laparoscopic LLS. ALT, Alanine Aminotransferase; AST, Aspartate Aminotransferase.

Early allograft dysfunction (EAD) occurred in 8 (17.4%) recipients. Vascular complications occurred in 4 cases (8.7%): portal vein stenosis in 1 (2.2%), portal vein thrombosis in 1 (2.2%, resulting in mortality), hepatic vein outflow obstruction in 1 (2.2%), and hepatic artery thrombosis (HAT) in 1 (2.2%, also resulting in mortality). The recipients with portal vein stenosis, hepatic vein stenosis, and biliary strictures were successfully managed with interventional therapy and recovered well. Biliary strictures developed in 3 recipients (6.5%), all of whom were treated by interventional radiology. Graft failure occurred in 3 cases (6.5%), including 1 retransplantation. Overall recipient mortality was 4.3% (2/46), with both deaths attributed to pre-existing severe vascular anomalies and high PELD scores (one due to intraoperative portal vein thrombosis and the other due to postoperative hepatic artery occlusion). No intraoperative dissection-related vascular injuries were observed in either donors or recipients (Tables 3, 4; Figure 2).

**Table 3:** Baseline characteristics of recipients.

Recipients	*N* = 46
Age (months)	13.3 ± 11.6
Sex (male/female)	27/19
Weight (kg)	7.7 ± 2.5
BMI	16.4 ± 3.1
Child-Pugh classification	
A	4 (8.7%)
B	24 (52.2%)
C	18 (39.1%)
PELD	16.1 ± 13.8
Diagnosis	
Cholestatic diseases	34 (73.9%)
Other cholestatic diseases	5 (10.9%)
Metabolic diseases	2 (4.3%)
Graft failure	3 (6.5%)
Hepatoblastoma	2 (4.3%)

PELD, pediatric end stage liver disease.

**Table 4:** Surgical outcomes of recipients.

Recipients	*N* = 46
GRWR (%)	3.0 ± 0.8
Anhepatic phase (min)	55.5 ± 9.5
Cold ischemia time (min)	157.4 ± 48.0
Operative time (min)	529.7 ± 123.0
Intraoperative blood loss (mL)	186.4 ± 173
PV anastomosis	
End-to-End	24 (52.2%)
Recipient Right-Left Bifurcation	17 (37.0%)
Venous bypass graft	4 (8.7%)
Recipient PV/Splenic vein bifurcation	1 (2.2%)
HV anastomosis	Piggyback technique
Donor Left HV—Recipient Left/Middle HV common orifice	43 (93.5%)
Segment III → Recipient Right HV (direct anastomosis)	2 (4.3%)
Reconstituted Segments II & III → Recipient HV (anastomosis after plasty)	1 (2.2%)
Hepatic artery anastomosis	
Single arterial	39 (84.8%)
Double arterial	7 (15.2%)
Biliary anastomosis	
Roux-en-Y	45 (98.8%)
Duct-to-Duct	1 (2.2%)
ICU length of stay (days)	5.5 ± 4.3
Total hospital stays (days)	30.0 ± 13.2
PV complications	
Stenosis	1 (2.2%)
Thrombosis	1 (2.2%, mortality)
HV complications	
Outflow obstruction	1 (2.2%)
Hepatic artery complications	
HAT	1 (2.2%, mortality)
Biliary complications	
Stricture	3 (6.5%)
Graft failure	3 (6.5%, 1 retransplantation)
Recipient mortality	2 (4.3%)

GRWR, graft recipient weight ratio; PV, portal vein; HV, hepatic vein; ICU, intensive care unit; HAT, hepatic artery thrombosis.

### 3.4 Literature comparison

Our outcomes were comparable with three major international laparoscopic LLS series (2, 5, 6). Donor operative time, blood loss, warm ischemia time, complication rates, and recipient outcomes were all within benchmark ranges (Table 5).

**Table 5:** Comparison of perioperative outcomes between present study and published laparoscopic LLS series.

Variables	Present study (*N* = 46)	Cherqui et al. (2021)	Hong et al. (2024)	Wei et al. (2020)
Donor operative time (min)	289.7 ± 66.7	268 ± 54	301 ± 62	312 ± 58
Donor blood loss (mL)	71.5 ± 60.3	65 ± 38	78 ± 49	85 ± 46
Warm ischemia time (s)	297.8 ± 98.6	<300	310 ± 95	320 ± 105
Donor complication rate	2.2%	2.8%	3.3%	3.9%
Donor vascular injury	0%	0%	0.7%	1.3%
Recipient biliary complication	6.5%	6.2%	5.8%	7.9%
Recipient vascular complication	8.7%	5.1%	4.7%	7.2%
Recipient mortality	4.3%	3.8%	4.1%	5.3%

LLS, left lateral sectionectomy.

## 4 Discussion

Major transplant centers worldwide have established a relatively standardized laparoscopic LLS procedure, which primarily consists of five key steps (5): (1) mobilization of the perihepatic ligaments; (2) management of the first hepatic hilum; (3) parenchymal transection; (4) management of the second hepatic hilum; and (5) graft extraction. However, significant controversy remains regarding the execution sequence of these steps, particularly between the “hilar-first” and “Parenchyma-First” strategies.

**Figure 3:**
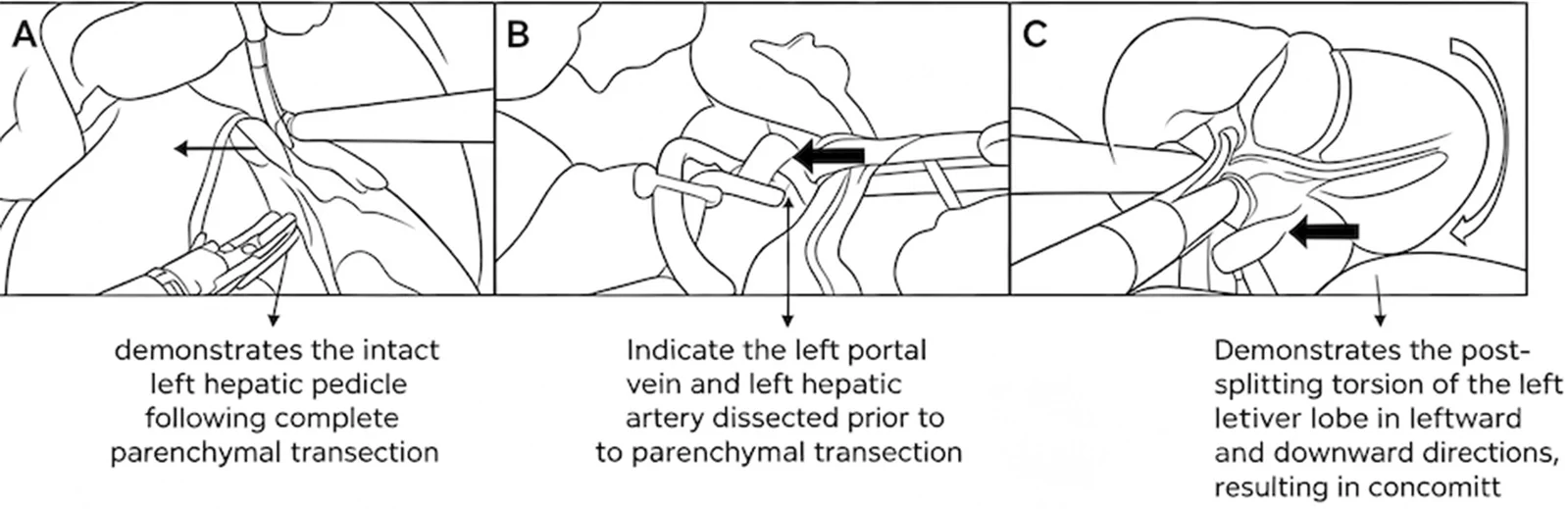
Surgical schematic by “Parenchyma-First” approach for laparoscopic LLS. **(A)** Fine arrows demonstrates the intact left hepatic pedicle following complete parenchymal transection. **(B)** Bold arrows indicate the left portal vein and left hepatic artery dissected prior to parenchymal transection. **(C)** The curved arrow demonstrates the post-splitting torsion of the left liver lobe in leftward and downward directions, resulting in concomitant compression, narrowing, and torsion of the left portal vein with consequent hemodynamic impairment.

The “Hilar-First” approach is grounded in a precise anatomical understanding of the hepatic vasculature. Surgeons traditionally first control hepatic inflow to identify the transection plane using the ischemic demarcation line (7). However, early skeletonization of the left hepatic artery and left portal vein deprives these vessels of structural support, rendering them vulnerable to traction or iatrogenic injury (4) (Figure 3). In particular, minor intimal injury to the hepatic artery may lead to severe graft arterial thrombosis postoperatively. In demanding left or right hepatectomies, these limitations may be magnified (8).

Hemodynamic alteration is another critical concern. Although many studies suggest that portal vein clamping during living donor hepatectomy has no significant adverse impact on recipient outcomes, our center considers that avoiding portal ischemia during graft procurement may be conducive to early graft functional recovery, especially in recipients with high PELD scores. Complete mobilization of the left portal vein may potentially alter its hemodynamic profile. Following parenchymal transection, the isolated left lateral lobe is theoretically prone to gravitational angulation or torsion of the portal vein, which could presumably compromise portal perfusion and potentially exacerbate ischemia-reperfusion injury (9).

To address these limitations, our team adopted the “Parenchyma-First” approach for laparoscopic LLS. The core modification is to perform parenchymal transection before hilar vascular dissection. Our series of 46 cases demonstrates that this modified strategy appears safe and feasible, accompanied by a low donor complication rate. By delaying hilar dissection until the final stage, the left portal vein and left hepatic artery may preserve their natural mechanical support and hemodynamic stability.

This approach offers several advantages: (1) Vascular protection: late hilar dissection minimizes mechanical trauma; (2) Hemodynamic preservation: the natural orientation and course of the portal vein are maintained; (3) Adaptive decision-making: anatomical variants can be assessed more accurately after substantial parenchymal division.

The present study has several limitations. First, it is a single-center, retrospective, non-controlled design without a hilar-first control group; thus, definitive superiority cannot be established. Second, formal learning curve analysis using the CUSUM method was not performed. Third, follow-up duration is relatively short for recent recipients, and survival analysis remains exploratory. Fourth, the parenchyma-first sequence is associated with a higher rate of multiple biliary orifices, increasing the complexity of recipient biliary reconstruction. Fifth, objective hemodynamic data (Doppler, pressure measurement) were not available to verify reduced torsion; the mechanistic rationale remains theoretical. Sixth, ICG guidance may have independently contributed to biliary outcomes (10, 11).

Our outcomes are comparable with major international laparoscopic LLS series (Table 3). Donor complication rate, vascular safety, warm ischemia time, and recipient survival are all within established benchmark ranges (6).

The 6.5% biliary stricture rate reflects a deliberate trade-off to maximize donor safety: we transected the bile duct more distally to protect the donor's biliary system, which resulted in multiple graft duct orifices. Although ICG fluorescence improved visualization, delayed hilar exposure inherently limits precise assessment of the bile duct (12, 13).

The Pringle maneuver was intentionally omitted to preserve physiological perfusion. Despite wide individual variability in blood loss, no donor required transfusion, supporting the safety of hemostasis without inflow occlusion.

Notably, no hepatic artery intimal injury occurred in any donor. The two recipient deaths were caused by severe pre-existing vascular abnormalities, not by the donor surgical technique.

## 5 Conclusion

The “Parenchyma-First” approach for laparoscopic living-donor LLS shows promise in terms of safety and feasibility and achieves good donor outcomes. This clinical adaptation, rather than a new surgical principle, could potentially reduce vascular trauma associated with LDLT-LLS.

Limitations include single-arm design, short follow-up and a higher prevalence of multiple bile duct orifices. Further controlled studies are needed to confirm its advantages.

## Data Availability

The raw data supporting the conclusions of this article will be made available by the authors, without undue reservation.
